# Effort Provision in a Game of Luck

**DOI:** 10.3389/fpsyg.2021.637339

**Published:** 2021-05-20

**Authors:** Mads Nordmo Arnestad, Kristoffer W. Eriksen, Ola Kvaløy, Bjørnar Laurila

**Affiliations:** ^1^Department of Leadership and Organizational Behaviour, BI Norwegian Business School, Oslo, Norway; ^2^University of Stavanger Business School, University of Stavanger, Stavanger, Norway

**Keywords:** effort (labor) costs, motivation, luck and chance, management, compensation

## Abstract

In some jobs, the correlation between effort and output is almost zero. For instance, money managers are primarily paid for luck. Using a controlled lab experiment, we examined under which conditions workers are willing to put in effort even if the output (and thus their employer’s earnings) is determined by pure luck. We varied whether the employer could observe the workers’ effort, as well as whether the employer knows that earnings were determined by luck. We find that, workers believed that the employer will reward their effort even if their effort does not affect earnings. Consequently, workers work harder if the employer could observe their (unproductive) effort. Moreover, even when the employer only saw earnings and not effort, workers labored harder if the employer did not know that earnings were determined by luck.

## Introduction

In most types of work, increased effort will lead to improved results. However, in some jobs, the relationship between effort and outcome is almost zero. For instance, the performance of many money managers is mostly a measure of luck (Malkiel and Fama, 1970; Fama and French, 2010; Pástor et al., 2017). A substantial body of research suggests that although some investors do outperform their relevant indexes, effort does not appear to set successful money managers apart from unsuccessful ones (Bhootra et al., 2015). In fact, money managers’ effortful behavior may even be negatively related to their performance. Firstly, transactional activity is negatively related to outcomes because transaction costs tend to outweigh the gains associated with these trades. Moreover, paying close attention to the market may result in erroneous reactions to non-predictive cues (Yates et al., 1991), and result in more frequent and more myopic transactions. This effect is prolific among both students and professional investors (Gneezy and Potters, 1997; Thaler et al., 1997; Gneezy et al., 2003; Haigh and List, 2005) and has been demonstrated in both lab and field experiments (Larson et al., 2016).

Among certain other jobs, the correlation between effort and performance can be quite small. Effort may be positively correlated with observable outcomes, but these outcomes are also a function of random events outside the workers’ control. Examples include professional “psychics” who provide predictions of their customers’ personal life. While this profession may seem to be on the fringe of the labor market, the market for psychic prediction suggests otherwise: In 2017, the term “psychic” ranked as the 12^th^ most expensive adword on Google (Guttmann, 2020). A survey of a French representative sample revealed that 19% of respondents had consulted a psychic or fortune-teller in their lifetime (Omnibus, 2018). Another example involves professional gamblers and sports betters, who approach gambling as a means of income rather than entertainment (Ladouceur and Walker, 1996). Estimates suggest that the number of professional gamblers in the United States is between 100,000 and 700,000 (Bluth, 1997). As there may be an element of skill in some forms of gambling (Ranyard and Charlton, 2006), many seasoned gamblers give an impression of being skilled (Delabbro and Winefield, 1999). However, studies show the relationship between effort and outcome is weak, and these skills are perhaps better understood as cognitive distortions (Cantinotti et al., 2004). Being knowledgeable within the gambling field may even be detrimental to performance, as knowledgeable players may respond to minor cues they think are predictive, which turn out not to be predictive (Ladouceur et al., 1998; Andersson et al., 2005). In general terms, any worker whose primary goal is to make accurate predictions of future events in settings where making such predictions is difficult or impossible is likely to be working under conditions where exerting more effort will have little impact on the outcome (Silver, 2012; Tetlock and Gardner, 2016).

Given the minimal and possibly negative relationship between effort and performance, it may appear that specifically money managers work little. However, evidence suggests they put substantial amounts of effort into their work in terms of both time and dedication (Michel, 2014). These two sets of seemingly incompatible observations lead us to pose the question. What motivates effort in a game of pure luck?

If effort is believed to be positively correlated with performance, and if high levels of noise (i.e., random variation) are compensated by high-powered incentives like tournament theory predicts (Lazear and Rosen, 1981), then people work hard even in a noisy environment where luck is important. However, what if the workers know their effort does not help performance? Will they still work hard? We investigated this question in a laboratory experiment by assessing under which conditions workers are willing to manifest effort, even if output (and thus the employer’s earnings) is determined by pure luck. We varied whether the employer, who rewarded the workers, can observe the workers’ effort, and whether the employer knows that earnings were determined by luck.

A growing body of literature offers evaluations of how people reward luck vs effort (Cappelen et al., 2013; see Cappelen et al., 2017). However, in contrast to these literature findings, our results provide information about the workers’ behavior and expectations, not the employers or impartial observers. Additionally, a few papers investigate the effect of noise on effort under various incentive systems (see Sloof and van Praag, 2010; Eriksen et al., 2011; Delfgaauw et al., 2013; Rubin and Sheremeta, 2015; Corgnet and Hernán-González, 2018). However, no research group has investigated effort provisions in environments where effort is completely unrelated to earnings and purely determined by luck.

## The Morality of Effort

Standard economic theory predicts that workers will not exert effort in any setting where effort is unrelated to outcome. However, we propose a moral psychology account to explain when and why people will exert effort in a game of pure luck. Moral psychology relies upon three normative ethical theories as a point of departure for moral judgment: consequentialist ethics, whereby the moral value of an action is evaluated on the basis of its material outcomes; deontological ethics, whereby the moral value of an action is judged on the basis of rules, duties, and obligations; and virtue ethics, in which the individual and not the action is the unit of moral evaluation (Uhlmann et al., 2015). Consequentialist theories of ethics hold that an act is permissible only if it maximizes good outcomes on quantifiable metrics. Typical examples include maximizing welfare and flourishing, minimizing resources spent, and maximizing lives saved (Smart and Williams, 1973). Consequentialist ethics provide little explanation for effortful work in a game of pure luck since the efforts of the worker are, by definition, unrelated to outcomes. Deontological ethical theories state that an action is right or wrong based on whether it violates a set of rules, duties, and obligations that are seen as foundational to morality (Kant, 1785). According to this view, an action can be wrong despite bringing about good consequences, and an action can be right despite bringing about bad consequences. Some actions may also be duty-bound even if they are unrelated to outcomes. In a game of luck, a worker should labor intensively if they adhere to a moral norm that states that hard work is an obligation in and of itself, regardless of its efficacy. Based on this assertion, we hypothesize that:

*(H1) Even when effort is unobservable to employers and unrelated to outcomes, most workers will choose to exert some effort.*

A worker may also be motivated to work hard if they expect the manager to adhere to a social norm of hard work. Under such conditions, the worker may expect that effort will be rewarded, irrespective of output. This latter point is related to the third ethical theory that informs moral judgment: virtue ethics. Virtue ethics is less concerned with evaluating actions and more concerned with evaluating people and whether they possess moral traits. A growing body of psychological research suggests that people intuitively and automatically make inferences about people’s moral traits (Todorov and Uleman, 2003; Willis and Todorov, 2006; Fiske et al., 2007). This tendency is observed across cultures (Choi and Nisbett, 1998; Lieberman et al., 2005) and even in young children (Hamlin et al., 2007). Observing effort is especially salient in judgments of virtue (Robinson et al., 2017), as effort influences people’s perception of the worker’s goals, intentions, and moral character (Pizarro and Tannenbaum, 2011; Uhlmann et al., 2015). According to Heider’s (1958) classical work, exertion of effort (i.e., how hard a person is trying to do something) signals a worker’s motivational force and the relative importance of the goal to the worker. Later research has supported the assertion that people infer goals from effortful behavior (Hassin et al., 2005). Many studies have highlighted the mediating role of the attribution of motivation in the judgment of moral character (Reeder and Spores, 1983; Reeder et al., 2002; Reeder, 2009). The more effort exerted by the worker, the more likely perceivers are to make inferences about the goal of the worker (Dik and Aarts, 2007). Furthermore, the more effort a worker exerts in pursuing a goal, the more people perceive that goal as important to the worker (Dik and Aarts, 2008; Bigman and Tamir, 2016). Thus, as long as the goal is seen as morally good, increased effort leads to improved judgments of moral virtue (e.g., Cushman, 2008). If it is “the thought that counts” (Rand et al., 2015) and effort is taken as a proxy for that thought, then workers should be motivated to work hard in a game of luck in order to demonstrate their virtue. We thus hypothesize that:

*(H2) When the employer can observe their efforts, workers will work harder than when employers do not observe efforts and expect to be rewarded for working hard.*

## Competing Predictions in Worker Ethics

Our first two hypotheses outline that both “inward-focused” deontological ethics and “outward focused” virtue ethics may motivate effort in a game of luck and that virtue ethics would provide the most powerful motivation. However, it is less obvious whether employees will work hard if the role of luck is common knowledge (i.e., when both the employer and the workers know that effort is unrelated to earnings). Under these conditions, it is possible to argue for competing predictions with regards to worker’s beliefs and consequent effort. On the one hand, a worker may believe the employer will be unimpressed with explicitly unproductive efforts. If this is the case, the worker may assume higher efforts will fail to elicit higher compensation from the employer. The worker may even suspect the employer will punish unproductive efforts and provide lower compensation for higher efforts, as the employer may view it as their job to correct misguided behavior through reductions in compensation. On the other hand, it is conceivable that workers will rely on a commonly held work ethic heuristic (see Kruger et al., 2004; Schrift et al., 2016) in which even explicitly unproductive efforts will be rewarded by the employer. Past research has demonstrated the link between effort and judgments about moral virtue is unrelated to outcomes (Bigman and Tamir, 2016). However, this effect has never been tested in a setting in which the lack of a relationship between effort and earnings is common knowledge. As a result, we hypothesize that workers will rely on the work ethic heuristic and expect that high effort will be rewarded, even when it is common knowledge that effort does not help performance. Therefore, we formulated our third hypothesis:

*(H3) Even when the lack of a relationship between effort and earnings is common knowledge, workers will expect employers to reward effort and consequently work hard.*

Lastly, we investigated the role of potential “undeserved rewards” in workers’ effort provisions. When effort is unobservable and the role of effort and luck is not known by the employers, workers may worry about being given “undeserved rewards” (i.e., rewards that the worker believes would not have been given if the employer had been informed about the lack of correlation between effort and luck). It is natural for the worker to believe that the employer, without receiving any prior information, would assume that whatever earnings are produced will at least be partly related to the worker’s effort level. If the earnings are substantial, it is natural for the worker to expect a substantial reward. This setting may be uncomfortable to some workers, who may feel negatively about being rewarded for earnings they did not cause. In this case, a form of inaction aversion could materialize (see Anderson, 2003) whereby workers work hard as a way to avoid the negative feeling of being rewarded for something they did not earn. Having exerted effort, even if it was unproductive, may make the reward more palatable to the employee. This leads to our fourth hypothesis:

*(H4) When effort is not observable to employers, workers will work harder when the employer is unaware of the lack of relationship between effort and earnings.*

## Materials and Methods

In a setting where output is purely random, we investigated how a worker’s decision to put in effort is affected by the effort’s observability. We also tested whether this decision to put forth effort is affected by the employer’s knowledge of the output’s cause.

There were two types of players in the experiment, namely workers and employers. The workers labored individually in pairings on behalf of the employer on a real effort task. For each worker, a random draw made by the computer determined the worker’s output from the working period. This was done after the working period ended so that the workers did not know their output before they started working. After the output was drawn, the output was converted into real money and added to the money from the other worker in the pairing. This sum was transferred to the employer, who was tasked with distributing two-thirds of the money between the two workers while keeping the last third for themselves. We explain below what information the employer had when making the distribution. The complete set of instructions can be found in Supplementary Appendix A.

**FIGURE 1:**
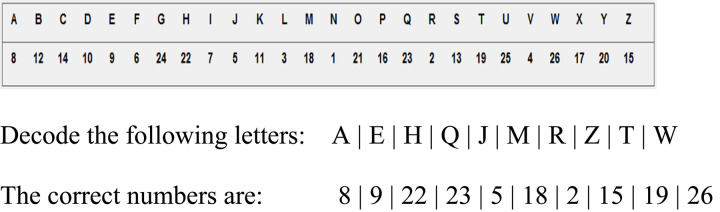
Example of real effort task.

The working period lasted for 20 min. The real effort task involved decoding a random string of 10 letters into a sequence of 10 numbers using a code sheet that listed the letters and their numbers. All numbers had to be correct in order to move to the next string, and there was an infinite number of strings for the worker to decode. Workers could decide the amount of effort they wanted to put in and decode as many or as few strings as they wanted. We used the number of strings a worker decoded as a measure of that worker’s effort. Figure 1 displays an example of the real effort task.

## Treatments

The workers always observed their own effort and were told that output was random. Workers were also informed about what information the employer received prior to the working period. Treatment variations were based on what information the employer had available when distributing the reward. The employer always viewed the individual worker’s output when making the distribution but knew only in two of the treatments that the output came from a random draw made by the computer. This allowed us to test the effect of both an informed and uninformed employer on workers’ effort. The other aspect we investigated was the effect of effort being observable. Therefore, we implemented two treatments in which the employer saw the individual worker’s effort and two treatments in which the employer did not see effort. Table 1 presents the design and treatments with the abbreviations used for the four treatments.

**TABLE 1:** Design and treatments.

	Luck known	Luck unknown
Effort observed	EOLK	EOLU
Effort unobserved	EULK	EULU

Our design seeks to distinguish between two main aspects that could motivate effort: effort observability and employer knowledge about the relationship (or lack of relationship) between effort and outcome. Thus, we employed a two-by-two design with the following four treatments. In the EOLK treatment (Effort Observable, role of Luck Known) effort was visible to the employer, and the role of luck was common knowledge. In EOLU treatment (Effort Observable, role of Luck Unknown) effort was also visible to the employer, but only the workers were told about the role of luck. In EULK treatment (Effort Unobservable, role of Luck Known) effort was not visible to the employer, while the role of luck was common knowledge. Finally, in EULU treatment (Effort Unobservable, role of Luck Unknown) effort was not visible for the employer, and only the worker was informed about the role of luck.

## Procedure

We ran 18 sessions in two batches with 255 participants in 2017, involving the first batch in June with 11 sessions and the second batch in August with seven sessions. Each session had a maximum of 23 participants and only one treatment. The participants were Norwegian-speaking students at the University of Stavanger who were recruited through an e-mail sent to all students at the university. Table 2 summarizes the characteristics of the participants in different conditions.

**TABLE 2:** Background characteristics.

	Age		Female		Grade		
			
Treatment	Mean	SE	Mean	SE	Mean	SE	N
Effort observed luck known (EOLK)	23.86	0.54	0.59	0.06	3.59	0.09	59
Effort unobserved luck unknown (EULU)	24.50	0.60	0.45	0.07	3.29	0.10	58
Effort observed luck unknown (EOLU)	24.59	0.95	0.57	0.07	3.36	0.07	56
Effort unobserved luck known (EULK)	24.31	0.89	0.64	0.06	3.22	0.08	64
All	24.21	0.38	0.57	0.03	3.36	0.04	237

*The table presents background characteristics of subjects in the experiment by treatment. “Age” is a variable measuring subjects’ age in years. “Female” notes the proportion of female participants. “Grade” measures self-reported average grade, ranging from 0 (= F) to 5 (= A).*

When the participants entered the lab, they drew a number from a jar that determined their place in the lab and subsequently their role; this also acted as a salient randomization device. An experimenter then read aloud general instructions about the rules, and the participants had 10 min to read the printed instructions carefully before the z-Tree (Fischbacher, 2007) program started. The written instructions contained information about the game, but these were not read aloud due to the nature of the treatments (see Supplementary Appendix A for full instructions). To verify that they had understood the instructions, workers had to answer correctly four true-or-false questions about the design. Then, their beliefs about how the employer would distribute rewards were elicited. The workers labored for 20 min. After the working period, workers saw their effort and learned their output based on what was drawn by the computer. When the employer made the distribution, we elicited the same beliefs as before the working period to test whether their beliefs had changed during the working period. We also asked the workers how they thought the employer should distribute the money. The responses to these questions were not incentivized.

There was only one employer in each session. This employer was asked to distribute the money between the two workers for all the pairs in that session. We decided to have only one employer in each session because the workers’ behavior was our primary interest. We did not communicate to the workers that one employer was responsible for all the pairs,^1^ only that they worked for a participant who was randomly selected to be an employer. When distributing the money, the employer saw the sums of money the workers had generated and, depending on the treatment, the number of strings each worker had decoded. The employer could not take money from one pair and distribute it to another pair; the whole amount of the two-thirds from the pair had to be distributed between the two workers in that particular pair for the computer to accept the distribution. After the employer had made all the distributions, the computer randomly selected one pair from which the employer received payment. The sessions lasted approximately 50 min, and the average payment was approximately NOK 230 (aprox 23 Euros).

## Results

This experiment consisted of both employers and workers, but the main research question pertains to what motivates effort when effort is unproductive; thus, the following main analysis focused on the beliefs and behavior of workers. We started by looking at workers’ beliefs, especially to what extent workers believe that effort will be rewarded by the employer. Although effort is unproductive in the sense that it does not affect earnings for the employer, it could affect earnings for the worker.

Table 3 presents participant responses to the following statement: “The employer will give the most money to the worker who has solved the most decoding tasks during the working period.” First, when the employer could observe effort, the majority of workers believed that effort would be rewarded. This was true irrespective of whether the employer was informed about the role of luck. By looking at the combination of columns 5 and 6 in Table 3, we find no significant differences in the frequency of subjects who believe that effort will be rewarded (or who are sure that effort will be rewarded), comparing EOLK (59.3%) and EOLU (64.3%) (test of equal proportions, *z* = −0.55, *p* = 0.58). In the two treatments where effort was not observable by the employer, the majority of subjects correctly believed the employer would not reward effort. According to row 3 and row 5 in Table 3, 93% (EULK) and 89% (EULU) of the subjects were neutral, did not believe effort would be rewarded, or were sure that effort would not be rewarded, respectively. While the responses in Table 3 for EULK and EULU indicate most workers understood that the employer cannot be affected by effort when deciding worker earnings, four workers in EULK and seven workers in EULU still indicated that they believed effort would be rewarded by the employer.^2^

**TABLE 3:** Belief about whether the employer will reward effort.

	0 = Sure it will not happen	1 = Do not believe it will happen	2 = Neutral	3 = Believe it will happen	4 = Sure it will happen	N
Effort observed luck known (EOLK)	0(0.00%)	10(16.95%)	14(23.73%)	30(50.85%)	5(8.47%)	59
Effort unobserved luck known (EULK)	34(58.62%)	11(18.97%)	9(15.52%)	3(5.17%)	1(1.72%)	58
Effort observed luck unknown (EOLU)	0(0.00%)	8(14.29%)	12(21.43%)	24(42.86%)	12(21.43%)	56
Effort unobserved luck unknown (EULU)	18(28.13%)	16(25.00%)	23(35.94%)	6(9.38%)	1(1.56%)	64
Total	52(21.94%)	45(18.99%)	58(24.47%)	63(26.58%)	19(8.02%)	237

*This table presents the frequencies (percentages) of responses to the statement: “The employer will give the most money to the worker who has solved the most decoding tasks during the working period.” The responses are measured on a Likert scale from 0 (sure that it will not happen) to 4 (sure that it will happen). The rightmost column gives the number of subjects.*

Table 4 supports the observations explained above. Using Mann-Whitney U-tests, we found that workers believed the employer would reward effort when effort was observable to the employer but not when effort was unobservable. Importantly, results suggest that when effort was observed by the employer, workers’ beliefs were insensitive to employers’ knowledge about the role of luck, as shown by the insignificant difference between EOLK and EOLU. Although the majority of workers believed that effort would not be rewarded in EULK and EULU, the workers tended to be more confident if the employer had knowledge about the role of luck compared to when the employer was uninformed about the role of luck. This finding is highlighted in rows 3 and 5 in Table 3, as more workers believed the employer would not reward effort in EULK compared to EULU. Also, the difference in responses was significant between EULK and EULU, as noted in Table 4.

**TABLE 4:** Comparison of belief about whether the employer will reward effort.

	EULU	EOLU	EULK
EOLK	5.90***	–1.23	7.48***
EULU		6.29***	–3.31***
EOLU			–7.62***

The table presents z-values from Mann-Whitney *U*-tests comparing responses to the statement: “The employer will give the most money to the worker who has solved most decoding tasks during the working period.” Responses are reported on a Likert scale from 0 (sure that it will not happen) to 4 (sure that it will happen). ***p < 0.001.

Next, we will discuss how workers actually behaved in the experiment (i.e., what effort they provided). First, workers demonstrated positive effort on average in all treatments despite their effort being unproductive.^3^ This observation is shown in Table 5, which presents a comparison of effort provision.^4^

**TABLE 5:** Comparison of effort provision.

	Luck known	Luck unknown	p-value
Effort observed	59.24	58.43	0.836
Effort unobserved	48.97	53.81	0.364
*p*-value	0.003	0.106	

*The table presents effort provisions in the experiments for each treatment. In addition, the table presents p-values from Mann-Whitney *U*-tests.*

A second observation highlights how workers labored harder if the employer could observe their effort, as compared to when the employer did not see effort. As shown in Table 5, effort provision when effort was observed and the role of luck was known (59.24) was significantly higher compared to effort provision when the role of luck was known by the employer, and effort was not observed by the employer (48.97). Qualitatively, a similar result was observed when comparing the two treatments in which the role of luck was not known to the employer, and effort was observed (58.43) or unobserved (53.81) by the employer. However, the difference is not significant, with a p-value of 0.106. Focusing only on the effect of effort observability, we combined the two treatments where the employer observed effort (EOLK and EOLU), comparing them to the two treatments where effort was not observed by the employer (EULK and EULU). This yielded a significantly higher effort when the employer could observe effort, with a mean effort of 58.84 compared to 51.51 for the combination of treatments where effort was not observed by the employer (Mann-Whitney *U*-test, *z* = −3.25, *p* = 0.001). Thus, effort provision was clearly affected by whether the employer could observe effort. However, even when effort was unobservable to employers, most workers chose to exert effort. This is consistent with social norms advocating that hard work is a moral duty and an obligation in and of itself.

Another observation, which was also consistent with the workers’ beliefs presented above, is that employers’ knowledge about the role of luck did not affect effort provision at all, as effort was observable. In Table 5, mean effort in EOLK was 59.24, while it was 58.43 in EOLU. This difference is not significant (Mann-Whitney *U*-test, *z* = 0.21, *p* = 0.84). Hence, even when the employer was informed that effort was unproductive (in the sense of not affecting earnings), workers still provided as much effort as when the employer only observed effort and did not know that the effort was unrelated to earnings. One interpretation of this finding is that effort provision is used as an important signaling device, informing the employer that the worker is deserving and a virtuous worker.

**FIGURE 2:**
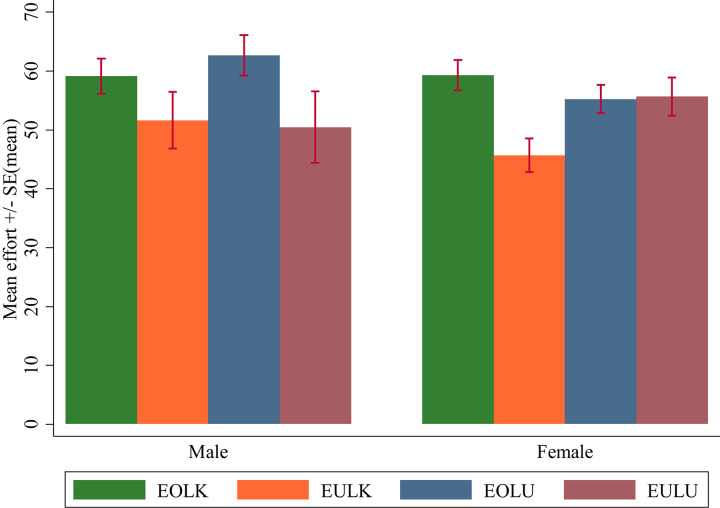
Mean effort by treatment and gender. The figure presents mean effort in the four treatments for both male and female subjects (error bars indicate standard errors of the mean).

In contrast, when effort was not observed by the employer, the role of luck seemed to matter. In EULK and EULU groups, effort was not observed by the employer, and Table 5 demonstrates that workers labored harder when the employer also did not know that earnings were determined by luck. However, while the mean effort in EULU was roughly 10% higher than in EULK, the difference is not statistically significant. Splitting our data by sex in Figure 2, we observe that effort provision from female participants was higher in EULU compared to EULK but not significant with *p* = 0.08 (Mann-Whitney *U*-test; 55.68 vs. 45.69; *z* = −1.74). In Supplementary Appendix B, we present summary statistics of effort for female participants (Supplementary Appendix Table 9) and corresponding tests of significance (Supplementary Appendix Table 10).

We ran a regression analysis to check the robustness of the results from the non-parametric test. Also, as noted in Table 2, the data regarding both gender and the subjects’ grades were not perfectly balanced.^5^ Thus, in the following regression analysis, we include the following: *Age*, which is a continuous variable measuring the subject’s age; *Female*, which is an indicator variable for participant gender; and *Grade*, which is an ordinal variable measuring self-reported average grade, ranging from 0 (= F) to 5 (= A). To indicate different treatments, we used two dummy variables: *Effort observed* is equal to one if the employer could observe the worker’s effort and is zero otherwise. *Luck unknown* is a dummy variable equal to one if the role of luck was unknown to the employer and is zero otherwise. Lastly, we include the interaction variable *Effort observed ^∗^ Luck unknown*. This interaction variable alone presents the difference-in-difference coefficient for our treatments (i.e., the difference in effort between EOLK and EULK and between EOLU and EULU). The reference group in our models consists of the condition where effort was unobserved by the employer but the employer was aware that workers were participating in a game of luck (EULK).

Regression results are found in Table 6. As indicated in model 3, *Grade* is positive and significant, showing workers with better grades tended to exert more effort, while *Age* is insignificant. More interesting are the dummy variables determining our treatments in model 1. *Effort observed* is positive and significant, demonstrating that workers exert significantly more effort when effort is observable by the employer. The coefficient for *Effort observed* alone gives the comparison of EOLK and EULK, while combining all coefficients (10.27 + 4.85 − 5.65 = 9.47) gives the comparison of EOLU versus EULK.^6^ Thus, controlling for worker’s age and academic performance, the regressions in Table 6 support the results from the previously presented non-parametric tests: workers worked harder if the employer could observe their unproductive effort.

**TABLE 6:** OLS regressions over effort provisions.

	(1)	(2)	(3)
Effort observed	10.272**(3.659)	10.545**(3.682)	9.005*(3.682)
Luck unknown	4.847 (3.587)	5.209 (3.626)	5.471 (3.583)
Effort observed * Luck unknown	−5.656(5.147)	−6.059(5.183)	−5.015(5.131)
Female		−1.884(2.623)	−1.955(2.590)
Age			−0.394(0.217)
Grade			4.331*(1.887)
Constant	48.966***(2.598)	49.810***(2.854)	40.897***(9.753)
R2	0.041	0.043	0.075
F	3.337	2.626	3.129
Observations	237	237	237

Effort observed, Luck unknown and Effort observed * Luck Unknown are dummy variables. Female is a dummy variable indicating participant gender. Age is measured in years. Grade is the subjects’ self-reported grade point average. Standard errors are in parentheses. *p < 0.05, **p < 0.01, ***p < 0.001.

Neither *Luck unknown* nor the interaction variable is significant, indicating that the employer’s knowledge about the role of luck was of less importance for workers when they had decided their effort provision. However, as indicated by Figure 2, male and female workers could differ in their response to whether the employer knew it was a game of luck. In Table 7, we investigate this further and present OLS regressions for male and female workers separately. Focusing on male workers first (model 5 and model 7), we see from the insignificant coefficient *Luck unknown* that no difference in effort provision existed between EULK and EULU. This was true when effort was observed and when we compare the two treatments with and without employer knowledge about the role of luck [EOLK≈59 and EOLU≈63, Wald test: *F*(1, 99) = 0.28, *p* = 0.60]. We also look at how effort observability affected male workers. As indicated by the coefficient *Effort observed*, the difference between EOLK and EULK is not significant. However, comparing the two treatments where effort was observed by the employer with the two treatments where effort was not observed by the employer yields a positive and significant effect [Wald test: *F*(1, 99) = 4.55, *p* = 0.04].

**TABLE 7:** Effort, sub-sample ols regression split for male/female participants.

	(4)	(5)	(6)	(7)
	Female	Male	Female	Male
Effort observed	13.622**(4.323)	7.500 (6.266)	12.796**(4.136)	6.734 (6.579)
Luck unknown	9.991*(4.186)	−1.147(6.344)	11.303**(4.008)	−0.989(6.461)
Effort observed * Luck unknown	−14.055*(5.848)	4.688 (9.226)	−13.260*(5.584)	4.961 (9.366)
Age			−0.543**(0.206)	0.003 (0.520)
Grade			5.822**(1.996)	1.720 (3.604)
Constant	45.692***(3.275)	51.625***(4.102)	32.952***(10.306)	44.283**(18.966)
*R*^2^	0.074	0.047	0.171	0.049
F	3.470	1.612	5.262	0.997
Observations	134	103	134	103

Effort observed, Luck unknown, and Effort observed * Luck unknown are dummy variables. Female is a dummy variable indicating participant gender. Age is measured in years. Grade is the subjects’ self-reported grade point average. Standard errors in parentheses. *p < 0.05, **p < 0.01, ***p < 0.001.

According to Model 4, female workers were significantly affected by whether the employer could observe effort. We did not hypothesize a gender difference at the outset of the experiment, but choose to report it here. In EOLK, female workers solved significantly more decoding tasks than they did in EULK, as shown by the positive coefficient of 13.62 for *Effort observed.* Also, female workers in EOLU solved close to 10 (13.62 + 9.99 – 14. − 5 = 9.56) more decoding tasks compared to those in EULK. This difference is also significant [Wald test: *F*(3, 130) = 3.47, *p* = 0.02].

Interestingly, the positive and significant coefficient for *Luck unknown* demonstrated female workers labored harder if they were paired with an employer who did not observe effort and also did not know the role of luck (EULK≈45 versus EULU≈45+10). This result indicates that female workers were sensitive to whether the employer knew the scenario was a game of luck. However, we only find evidence for such an effect when effort was unobserved by the employer. When the employer could see effort provision, the effort-observability effect seems to dominate, and we observe no significant difference between the treatments where the employer was informed or not informed about the role of luck [EOLK≈59 and EOLU≈55, Wald test: *F*(1, 130) = 0.99, *p* = 0.32]. One interpretation of this result is that effort observability was less effective for female workers when the employer did not know the scenario was a game of luck. This suggests female workers may labor harder compared to other groups in order to avoid undeserved rewards. Another way of looking at this is by the significant interaction term, which indicates the difference in effort between EOLK and EULK diverges from the difference in effort between EOLU and EULU.

## Discussion

Our experimental results provide support for all four hypotheses:

1. Most subjects exerted positive effort even when effort was unproductive.
2. They exerted more effort when effort was observable.
3. They expected employers to reward effort even if the employers knew output was determined by luck.
4. In the case where effort was unobservable, subjects worked harder if the employer did not know earnings were determined by luck.

The latter results were driven by female workers, reflecting past research suggesting that females place an overall higher personal value on effort (McCrea et al., 2008). It is important to note that we did not expect a gender difference at the outset of the experiment. As such, there is a relevant chance that the observed relationship reflects a random effect. However, we find that the result ties in with a greater stream of research indicating that female research participants demonstrate a stronger general tendency to portray themselves in a socially desirable manner (see Dalton and Ortegren, 2011).

To the best of our knowledge, these results are novel. The effect of noise on effort provision has been explored before, but no past studies have looked at effort provision in a setting where the correlation between effort and outcome is zero. Similarly, the relationship between observable effort and judgments of character has been explored numerous times but never in a setting where the futility of effort is common knowledge. Even in cases where effort was completely unrelated to outcomes, participants in this study tended to obey a work ethic heuristic. This was especially true when effort was observable, suggesting the work ethic heuristic has less to do with outcomes and more to do with social signaling. Our participants also expected to be rewarded for effort, even if the lack of relationship between effort and outcomes was common knowledge. This implies our participants expected that the work ethic heuristic was shared among their peers and that those who followed it would be rewarded for doing so, regardless of the outcome. While all participants exerted effort as an outward social signal when effort was observable, female participants also exerted effort as an inward social signal by working hard even when effort was unobservable.

There are some other possible reasons why the research participants chose to exert unproductive effort. Experimenter demand-effect may have prompted some of the participants to work. Similarly, boredom could be a motivating factor. While we cannot rule out these factors completely, we nevertheless believe that their role in the observed relationships is limited. Firstly, the demand effect or boredom effect would have been equal across treatments. Secondly, the participants were told that they were allowed to use their phones when they had finished working. As such, they would most likely have found alleviation from boredom more effectively by surfing the web rather than working at a mindless task which was explicitly unrelated to outcomes.

We instead interpret our results in the light of a work-ethic heuristic; the simplified view that effort is always preferable to less effort. As a general rule in life, people will observe that effort is related to outcomes, and outcomes are related to rewards. As such, most adults will approach any novel task with an implicit understanding that their performance can be improved with effort, and that good performances will be rewarded. This relationship is further cemented by cultural norms and practices that elevate the moral value of hard work, and condemn the sin of sloth and inactivity. The combined effects of cultural norms and intra-personal learning makes people behave in a way that is consistent with a work-ethic heuristic. In our experiment, however, effort was unrelated to performance. This demonstrates that the work-ethic heuristic, like most heuristics, is useful and adaptive in the normal set of circumstances, but lead to unproductive behaviors in different circumstances. As a general rule, reliance on the heuristic is beneficial at both the individual, organizational and societal level. However, in the few but notable cases where effort is unrelated to outcomes, the consequence of continued reliance on the work-ethic heuristic depends on the perceived cost of effort. If the workers experienced cost of effort is negative, reliance on the work-ethic heuristic will still produce a favorable outcome. However, if the experienced cost of effort is positive, as we argue it was in our experiment, continued reliance on the work-ethic heuristic leads to waste of resources.

Our experimental design is rather stylized. In the real world, neither workers nor employers will have full knowledge about the relationship between effort and output, and they will typically hold beliefs that effort—to some extent or in some cases—leads to higher performance. However, these lab experiments offered the advantage of an environment where only luck mattered and where we could control whether and to whom this information was available. This helps rule out confounding factors that may matter in real world environments where luck is important but not definitively. Additionally, it allows us to rule out standard economic theory as potential explanations for the results we achieved.

## Conclusion

This paper presents results from a controlled lab experiment investigating under which conditions workers were willing to put in effort, even if output (and thus employer’s earnings) was determined by pure luck. We varied whether the employer could observe the workers’ effort, as well as whether the employer knew that earnings were determined by luck. Standard economic theory predicts that workers would not exert effort in any of the conditions we investigated. However, we propose a form of moral psychology can explain when and why people will exert effort in a game of pure luck, namely deontological ethics (whereby the moral value of an action is judged on the basis of rules, duties, and obligations) and virtue ethics (in which the individual, not the action, is the unit of moral evaluation).

This experiment yielded the following results. First, subjects exerted positive effort even when this effort was unproductive. Second, subjects exerted more effort when the unproductive effort was observable than when it was not. Third, subjects expect employers to reward effort even if participants knew that output was determined by luck. Fourth, when effort was unobservable, subjects worked harder if the employer did not know that earnings were determined by luck.

## Data Availability Statement

The raw data supporting the conclusions of this article will be made available by the authors, without undue reservation.

## Ethics Statement

Ethical review and approval was not required for the study on human participants in accordance with the local legislation and institutional requirements. The patients/participants provided their written informed consent to participate in this study.

## Author Contributions

All authors listed have made a substantial, direct and intellectual contribution to the work, and approved it for publication.

## Conflict of Interest

The authors declare that the research was conducted in the absence of any commercial or financial relationships that could be construed as a potential conflict of interest.

## Footnotes

1 Excerpt from workers’ instructions: “There are two types of players in this experiment, employers and workers. You have been randomly selected to be a worker” and “A third participant has been randomly chosen to be an employer.” “You and the other worker are invited to work individually on a task on behalf of the employer.” See Supplementary Appendix A for full instructions.

2 We can only speculate why these subjects’ held beliefs that were at odds with the information given about the employer’s information. However, for this analysis, we include these workers if not stated otherwise.

3 We also allowed for alternative activities (e.g., internet surfing), and the instructions explicitly stated, *“*You choose how to spend your time in the experiment. Nevertheless, we require that you stay in the designated space throughout the experiment. You can use your mobile phone to browse the internet, but make sure it is in silent mode before we start.”

4 In Supplementary Appendix Table 8, we present more detailed summary statistics for each treatment. This table shows some workers (only four in total) chose to refrain from providing effort, and these workers all participated in treatments where effort was not observed by the employer.

5 See Supplementary Appendix Table 11 for non-parametric tests of differences in the background variables between treatments.

6 An *F*-test on whether the coefficients are jointly equal to zero is rejected [Wald test: *F*(3, 233) = 3.34, *p* = 0.02].
