# Supplementary material for: Effort Provision in a Game of Luck
Source: Front Psychol. 2021 May 20;12:637339. doi: 10.3389/fpsyg.2021.637339 (PMC8174658; doi:10.3389/fpsyg.2021.637339)
Supplement: Supplementary file 1 [file Data_Sheet_1.docx]

Appendix A:

*Below are the full written instructions for both workers and employers. Treatment specific instructions are in bold.*

### [Common welcoming text for all participants and treatments.]

Welcome to the Experiment!

Introduction

The experiment will last for about 45 minutes. During the experiment you will be able to earn money that will be transferred to your bank account when the experiment is over. It is therefore important that you enter the correct account number at the end of the experiment. If you do not have a Norwegian account number, please contact us after the experiment is completed. Instructions for the experiment can be found in this instruction manual. You will also get a summary of the instructions on the PC screen when the experiment itself starts.

This experiment is part of a research project and it is important for us that the following rules are followed:

- If you have questions, please raise your hand and we will come help you.
- Do not ask questions in plenary.
- It is not allowed to communicate with the other participants while the experiment is in progress.
- You choose how to spend your time in the experiment. Nevertheless, we require that you stay in the designated space throughout the experiment.
- You can use your mobile phone to browse the internet, but make sure it is on silent mode before we start.
- It is strictly forbidden to use your PC for anything other than the experiment, as other uses may lead to technical problems with the experiment.

You will now have time to read through the instructions for the experiment. Are there any questions about what has been said up to now?

Please turn to the next page.

Good luck!

### [Worker Instructions]

In this experiment, there are two types of participants, workers and employers. You are randomly drawn the role of a **worker**. Together with another randomly drawn participant, you form a worker pair. A third participant is randomly drawn the role of employer. You will not know the identity of either your fellow employee or employer, nor will they know your identity.

The task

You and the other worker are invited to work separately with a work assignment on behalf of the employer. The assignment consists of decoding letters to numbers. The PC screen will display a table of letters and corresponding numbers. Your job is to find the number in the table that matches the letters to be decoded (see example below). Once you have answered correctly on a task, the next task will appear on the screen. The working period is 20 minutes. Along the way, a clock in the top right corner will show how many seconds remain (20 minutes = 1200 seconds).

Example: Given the following table


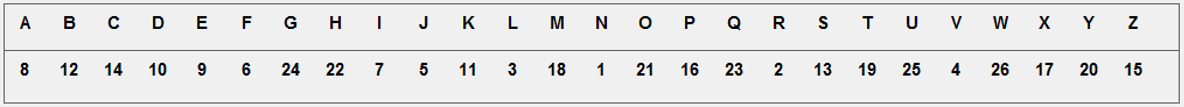


Decode the following letters: A | E | H | Q | J | M | R | Z | T | W

The correct answer is: 8 | 9 | 22 | 23| 5 | 18| 2 |15|19 | 26

When the work period is over, you will know how many decoding tasks you have solved.

Payment

When the work period is complete, the computer will draw a random number between 1 and 6, where each number is equally likely. In other words, the computer throws a die. It is this die and only this die, which determines how much money you earn / generate for your employer. If your computer throws a high number, you earn a lot of money for your employer, while low numbers earn less money for your employer. You will know your die throw after the working period and at the same time you will see how many letters you decoded during the work period.

Likewise, the computer will also roll a die for the other worker. The employer thus receives money from you and from the other worker. How much money the employer receives in total will depend on the two dice thrown.

The employer will retain 1/3 of the money you and the other worker generate. The remaining 2/3 of the money will be distributed by the employer between you and the other worker. Employers can freely choose the amount of money that will go to you and how much will go to the other worker.

### [EOLK: Effort is visible to everyone, and cause of output is common knowledge.]

**Before the employer decides how he / she will allocate the money between you and the other worker, he / she will see how many decoding tasks each of you solved and how many eyes on the die each of you got. The employer is aware that the amount you have earned / generated is due to the die, and not the effort in the task before the die is thrown. The employer also knows you have learned that how much money you generate for him / her is due to the die and not how many decoding tasks you solved during the working period.**

**The employer thus has the following information when distributing the money between you and the other worker:**

- **How many decoding tasks you have solved during the working period.**
- **How many decoding tasks the other worker has solved during the working period.**
- **How much money (eyes on the die) you generated for the employer.**
- **How much money (eyes on the die) the other worker generated for the employer.**
- **The employer knows that it is only the random dice that determine how much money is generated and that you and the other worker are also aware of this.**

#### *[EULK: Only workers see their effort, Cause of output is common knowledge.]*

**Before the employer chooses how he / she will allocate the money between you and the other worker, he will see which die each of you got. He will not see how many decoding tasks either you or the other worker solved.**

**The employer is aware that the amount you have earned / generated is due to the die, and not the effort in the task before the die is thrown. The employer also knows you have learned that how much money you generate for him / her is due to the die and not how many decoding tasks you solved during the working period.**

**The employer thus has the following information when distributing the money between you and the other worker:**

- **How much money (eyes on the die) you generated for the employer**
- **How much money (eyes on the die) the other worker generated for the employer**
- **The employer knows that it is only the random dice boxes that determine how much money is generated and that you and the other worker are also aware of this.**
- **The employer does not know how many decoding tasks you solved during the work period.**
- **The employer does not know how many decoding tasks the other worker solved during the working period.**

#### *[EOLU: Effort is visible to everyone, Only workers know the cause of output.]*

**Before an employer chooses how he / she will distribute the money between you and the other worker, he / she will see how many decoding tasks each of you has solved and how many eyes on the dice each of you got.**

**The employer does not know that the amount you have earned / generated is due to the dice, and not the effort in the task before the dice are thrown. Employers also do not know you have learned that how much money you generated for him / her is solely due to the dice, and not how many decoding tasks you have solved during the work period.**

**The employer has the following information when distributing the money between you and the other worker:**

- **How many decoding tasks you have solved during the working period.**
- **How many decoding tasks the other worker has solved during the working period.**
- **How much money (eyes on the die) you generated for the employer.**
- **How much money (eyes on the die) the other worker generated for the employer.**
- **The employer does not know that it is only the random dice throws that determine how much money is being generated, nor that you and the other worker are aware of this information.**

#### *[EULU: Only workers see their effort, Only workers know the cause of output.]*

**Before the employer chooses how he / she will allocate the money between you and the other worker, he will see which dice each of you got. He will not see how many decoding tasks you or the other worker solved.**

**The employer does not know that the amount you have earned / generated is due to the dice, and not the effort in the task before the dice are thrown. Employers also do not know you have learned that how much money you generated for him / her is solely due to the dice, and not how many decoding tasks you have solved during the work period.**

**The employer thus has the following information when distributing the money between you and the other worker:**

- **How much money (eyes on the die) you generated for the employer**
- **How much money (eyes on the die) the other worker generated for the employer**
- **The employer does not know how many decoding tasks you solved during the work period**
- **The employer does not know how many decoding tasks the other worker solved during the working period**
- **The employer does not know that it is only the random dice throws that determine how much money is being generated, nor that you and the other worker are aware of this information.**

Question and summary

Both before and after the work period you will be asked to answer some questions. Please try to answer as well as you can.

Once you have done this, you will get a screen that summarizes the outcome of the experiment. You will again see how many decoding tasks you have solved during the work period, which dice the computer threw for you after the work period, and how much you have earned in this experiment (the amount assigned to you by the employer). This section of the experiment may take some time, so please be patient.

Finally, you will be asked to answer a single questionnaire on your PC. This questionnaire asks you, among other things, to enter your account number. Be sure to fill in the correct account information so that you will receive the proceeds from the experiment. If you do not have a Norwegian account number, please contact one of us.

Good luck!

### [Employer instructions]

In this experiment there are two types of participants, workers and employers. You are randomly drawn the role of **employer**. The other participants in the experiment are randomly drawn to be workers. The workers are assembled in anonymous worker couples. You will not know the identity of the workers and they will not know your identity.

The workers’ task

In the experiment, there are several worker couples who all have you as an employer. Workers are invited to work individually with a work assignment on your behalf. The work assignment consists of decoding letters to numbers. The working period is set to 20 minutes. In this 20-minute period you have no tasks. You can use your mobile to browse the internet, but make sure it is on silent mode.

#### *[EOKL: Effort is visible to everyone and cause of output is common knowledge.]*

**For each worker couple:**

**When the work period is completed, the computer will draw a random number between 1 and 6, where each number is equally likely to be drawn. The computer makes such a draw for each worker. This is, in other words, as if the computer throws a die. Only the dice determine how much money the workers earn for you. If the computer draws a high number, they earn a lot of money for you while low numbers earn you less money. Specifically, each eye on the die equates to 100 NOK (1 eye = 100 NOK, 2 eyes = 200 NOK, etc.). After the working period, you will learn how much money each worker has earned (dice thrown) and how many decoding tasks each worker has solved.**

Payment and your task

You will receive 1/3 of the money each worker-couple earns. Your task will be to distribute the remaining 2/3 of the money that each worker pair earns between the two workers. You choose completely freely how much money you will give to each of the two workers. This must be done for all worker pairs. For all workers, this is real money and the only profit in the experiment.

IMPORTANT: After you allocate the money between the two workers for all the pairs, the computer will draw a random worker pair that determines your profits in the experiment. Your profit in the experiment is thus 1/3 of the money for a randomly drawn worker pair (not the sum of all worker pairs).

**To make the distribution, you will be given the following information for each worker pair:**

- **How many decoding tasks each worker solved during the working period**
- **How much money (eyes on the die) each worker generated for you**
- **Workers know that you have this information.**

#### *[EULK: Only workers see their effort and cause of output is common knowledge.]*

**For each worker couple:**

**When the work period is completed, the computer will draw a random number between 1 and 6, where each number is equally likely to be drawn. The computer makes such a draw for each worker. This is, in other words, as if the computer throws a die. Only the dice determine how much money the workers earn for you. If the computer draws a high number, they earn a lot of money for you while low numbers earn you less money. Specifically, each eye on the die equates to 100 NOK (1 eye = 100 NOK, 2 eyes = 200 NOK, etc.). After the working period, you will learn how much money each worker has earned (dice thrown).**

Payment and your task

You will receive 1/3 of the money each worker-couple earns. Your task will be to distribute the remaining 2/3 of the money that each worker pair earns between the two workers. You choose completely freely how much money you will give to each of the two workers. This must be done for all worker pairs. For all workers, this is real money and the only profit in the experiment.

IMPORTANT: After you allocate the money between the two workers for all the pairs, the computer will draw a random worker pair that determines your profits in the experiment. Your profit in the experiment is thus 1/3 of the money for a randomly drawn worker pair (not the sum of all worker pairs).

**To make the distribution, you will be given the following information for each worker pair:**

- **How much money (eyes on the die) each worker generated for you**
- **Workers know that you have this information.**

#### *[EOLU: Effort is visible to everyone and only workers know the cause of output.]*

**For each worker couple:**

**You as an employer will know how many decoding tasks each worker has solved during the working period and how much money each worker has earned for you.**

Payment and your task

You will receive 1/3 of the money each worker-couple earns. Your task will be to distribute the remaining 2/3 of the money that each worker pair earns between the two workers. You choose completely freely how much money you will give to each of the two workers. This must be done for all worker pairs. For all workers, this is real money and the only profit in the experiment.

IMPORTANT: After you allocate the money between the two workers for all the pairs, the computer will draw a random worker pair that determines your profits in the experiment. Your profit in the experiment is thus 1/3 of the money for a randomly drawn worker pair (not the sum of all worker pairs).

**To make the distribution, you will be given the following information for each worker pair:**

- **How many decoding tasks each worker solved during the working period**
- **How much money (eyes on the die) each worker generated for you**
- **Workers know that you have this information.**

#### *[EULU: Only workers see their effort and only workers know the cause of output.]*

**For each worker couple:**

**You as an employer will know how much money each worker has generated for you.**

Payment and your task

You will receive 1/3 of the money each worker-couple earns. Your task will be to distribute the remaining 2/3 of the money that each worker pair earns between the two workers. You choose completely freely how much money you will give to each of the two workers. This must be done for all worker pairs. For all workers, this is real money and the only profit in the experiment.

IMPORTANT: After you allocate the money between the two workers for all the pairs, the computer will draw a random worker pair that determines your profits in the experiment. Your profit in the experiment is thus 1/3 of the money for a randomly drawn worker pair (not the sum of all worker pairs).

**To make the distribution, you will be given the following information for each worker pair:**

- **How much money (eyes on the die) each worker generated for you**
- **Workers know that you have this information.**

## Appendix B

*Table 8 Summary Statistics: Effort provisions*

|  | **Mean** | **SD** | **Median** | **Min** | **Max** | **N** |
| --- | --- | --- | --- | --- | --- | --- |
| **Effort observed luck known (EOLK)** | 59.2 | 14.8 | 55 | 33 | 95 | 59 |
| **Effort unobserved luck unknown (EULU)** | 49.0 | 22.5 | 48 | 0 | 128 | 58 |
| **Effort observed luck unknown (EOLU)** | 58.4 | 15.4 | 55 | 28 | 97 | 56 |
| **Effort unobserved luck known (EULK)** | 53.8 | 24.1 | 53 | 0 | 125 | 64 |
| **Effort observed** | 58.8 | 15.0 | 55 | 28 | 97 | 115 |
| **Effort unobserved** | 51.5 | 23.4 | 51 | 0 | 128 | 122 |
| **Luck known** | 54.1 | 19.7 | 54 | 0 | 128 | 117 |
| **Luck unknown** | 56.0 | 20.5 | 54 | 0 | 125 | 120 |

Note: The table presents summary statistics of effort provisions in the experiment for each treatment. In addition, we also include combinations of treatments. Here *Effort observed (Effort unobserved)* consists of the two treatments where effort is observable (unobservable). *Luck known (Luck unknown)* consists of the two treatments where the role of luck was known (unknown) to the employer.

*Table 9 Summary Statistics: Effort provisions - Females*

|  | **Mean** | **SD** | **Median** | **Min** | **Max** | **N** |
| --- | --- | --- | --- | --- | --- | --- |
| **Effort observed luck known (EOLK)** | 59.3 | 15.2 | 55 | 38 | 95 | 35 |
| **Effort unobserved luck unknown (EULU)** | 45.7 | 14.6 | 46.5 | 0 | 69 | 26 |
| **Effort observed luck unknown (EOLU)** | 55.3 | 13.5 | 54.5 | 28 | 95 | 32 |
| **Effort unobserved luck known (EULK)** | 55.7 | 20.8 | 54 | 14 | 125 | 41 |
| **Effort observed** | 57.4 | 14.5 | 55 | 28 | 95 | 67 |
| **Effort not observed** | 51.8 | 19.2 | 52 | 0 | 125 | 67 |
| **Luck known** | 53.5 | 16.3 | 52 | 0 | 95 | 61 |
| **Luck unknown** | 55.5 | 17.9 | 54 | 14 | 125 | 73 |

Note: The table presents summary statistics of effort provisions in the experiment for each treatment, by female. In addition, we also include combinations of treatments. Here *Effort observed (Effort unobserved)* consists of the two treatments where effort is observable (unobservable). *Luck known (Luck unknown)* consists of the two treatments where the role of luck was known (unknown) to the employer.

*Table 10 Mann-Whitney U-tests Effort provided – Females*

|  | **EULU** | **EOLU** | | | | **EULK** | | **Effort unobserved** | | | | | **Luck unknown** |  |  |  |
| --- | --- | --- | --- | --- | --- | --- | --- | --- | --- | --- | --- | --- | --- | --- | --- | --- |
| **EOLK** | 1.06 | | 0.81 | | | 2.85** | | | | |  | | | |  | |
| **EULU** |  | 0.14 | | | | -1.74 | | | | |  | | | |  | |
| **EOLU** |  | | | |  | -2.38* | | | | |  | | | |  | |
| **Effort observed** |  | | | |  | |  | | | | -2.06* |  | | | |  |
| **Luck known** |  | | |  | | | | |  |  | | | 0.54 |  |  |  |

Note: The table presents z-values from Mann-Whitney U-tests. *:p<0.05, **:p<0.01, ***:p<0.001. In addition, we also include combinations of treatments. Here Effort observed (Effort unobserved) consists of the two treatments where effort is observable (unobservable). Luck known (Luck unknown) consists of the two treatments where the role of luck was known (unknown) to the employer.

*Table 11 Comparison of background characteristics*

| **Age**  **Female**  **Grade** | |  |  |
| --- | --- | --- | --- |
|  | **Effort unobserved luck unknown** | **Effort observed luck unknown** | **Effort unobserved luck known** |
| **Effort observed luck known** | 1.00  -0.54  3.00** | 0.32  0.24  2.00* | -0.62  1.57  2.17* |
| **Effort unobserved luck unknown** |  | 0.38  -0.77  1.28 | 1.54  -2.13*  0.58 |
| **Effort observed luck unknown** |  |  | 0.90  -1.31  -0.52 |

Note: The table presents z-values from Mann-Whitney U-tests (Age and Grade) and tests for the equality of proportions (Female), comparing Age/Female/ Grade by treatment. "Age" is a variable measuring subjects age in years; "Female" presents the proportion of females; "Grade" measures self-reported average grade, ranging from 0(=F) to 5 (=A). *:p<0.05, **:p<0.01, ***:p<0.001.
